# Magnitude of enteric pathogens associated with diarrhea and antibiotic resistance of enteric bacterial pathogens isolated among children under 5 years of age in Bule Hora town, West Guji, Ethiopia

**DOI:** 10.3389/fpubh.2024.1398264

**Published:** 2024-10-07

**Authors:** Girma Ashenafi, Dagnamyelew Tilahun, Alqeer Aliyo, Biruk Sisay

**Affiliations:** Department of Medical Laboratory Sciences, Institute of Health, Bule Hora University, Bule Hora, Ethiopia

**Keywords:** diarrhea, antimicrobial susceptibility, enteric bacteria, gastroenteritis, Bule Hora

## Abstract

**Background:**

Diarrhea is the second leading cause of morbidity and mortality worldwide among all ages, and one of the leading causes of poor health and premature death in the developing world. Microorganisms, such as viruses, bacteria, and parasites, are responsible for enteric infections among children. Excessive and inappropriate use of antimicrobial drugs and poor infection control practices have transformed antimicrobial resistance into a serious threat to public health worldwide. Therefore, it is essential to investigate the prevalence of enteric pathogens and the antimicrobial susceptibility patterns of bacterial pathogens.

**Objective:**

Assess the prevalence of enteric pathogens associated with diarrhea in children under 5 years at the Bule Hora Health Facility in 2021.

**Methods:**

An institution-based cross-sectional study was conducted from May 2021 to July 2021 in a Bule Hora Health Centre and Hospital. A total of 422 children with diarrhea were included in the study in the outpatient department. Sociodemographic and associated factors were evaluated using a pre-tested questionnaire. A sufficient amount of stool specimens was collected following standard microbiological procedures. An antigen detection kit was used to diagnose rotaviruses, parasites were examined using microscopy, and bacterial identification was carried out by culture and biochemical tests. The antibiotic susceptibility test of the bacterial isolates was performed using the Kirby-Bauer disc diffusion method. The data were analyzed using SPSS version 20. The *p*-value less than 0.05 was declared statistically significant.

**Results:**

The overall prevalence of enteropathogens in children under 5 years of age was 17%, with no mixed infections detected. Of this, 7.8% of the children’s stool samples contained bacterial pathogens, 5% tested positive for rotavirus, and 4.2% contained intestinal parasites. Unprotected drinking water sources, poor carrier hand washing practices, and poor cleaning of utensils for child feeding were factors significantly associated with the prevalence of enteropathogens. Bacterial isolates have shown a high prevalence of resistance to amoxicillin.

**Conclusion:**

Therefore, it is important to take steps to reduce the spread of enteric pathogens among children under 5 years practicing good hygiene, ensuring sanitation, and providing clean drinking water. We recommend performing antimicrobial susceptibility tests before prescribing treatment to children with diarrhea.

## Introduction

Diarrhea is the second leading cause of morbidity and mortality worldwide among all ages, and one of the leading causes of ill health and premature death in the developing world (1, 2). About 72.8 million people are exposed to diarrhea disability per day (3). Common bacterial agents that cause diarrhea include *Salmonella* spp., *Shigella* spp., *Campylobacter jejun*, *Clostridium difficile*, *Aeromonas* spp., *Plesiomonas* spp., *Enterobacter* spp., *S. aureus*, *K. pneumoniae*, *Morganella morgani*, *and E. coli* is the most common cause of moderate to severe diarrhea in the first 2 years of life (1, 4). The most common parasites that cause diarrhea include *Ascaris lumbricoides*, *Trichuris trichiura*, *Hookworm*, *Hymenolopses nana*, *Entameoba histolytica/disar*, and *Giardia lamblia* (5).

Intestinal tract infections ranging from mild to severe are caused by Shigella and Salmonella. The consumption of tainted food and water is how they spread. These infections are common in developing countries where fly, lack of a suitable sewage treatment system, and lack of clean water supplies contribute to the development of the disease (6, 7). *Escherichia coli*, *Campylobacter*, and *Vibrio* spp. are involved in the epidemiology of diarrhoea, especially in some parts of the world (4, 7). In most industrialized and developing nations, the rotavirus has been identified as the primary enteric pathogen in children (8).

Diarrheal disease is associated with the occurrence of the disease in sub-Saharan African nations, including Ethiopia, mainly as a result of low levels of education, inadequate access to safe drinking water, poor sanitation and hygiene, and generally lower levels of general health and nutritional condition (9, 10).

Bacterial antibiotic resistance has become a widespread and well-known phenomenon, and this resistance continues to accumulate with multidrug resistance to commonly prescribed antibiotics, including cotrimoxazole, ampicillin, tetracycline, and amoxicillin (11, 12). Today, especially in our country, where there are only a few established bacteriological laboratories, enteric pathogens are treated mainly without an investigation of antimicrobial susceptibility, resulting in increased drug resistance to commonly used antibiotics (12, 13). We are approaching a post-antibiotic future where routine diseases and small injuries can once again be fatal if we do not take immediate action (14). Global estimates indicate that diseases resistant to current medications cause the deaths of about 200,000 babies annually; the great majority of these deaths occur in underdeveloped countries (15, 16).

Therefore, it is important to explore the prevalence of enteric pathogens among children under 5 years of age. Furthermore, understanding the antimicrobial susceptibility pattern of bacterial isolates will help clinical professionals make informed decisions when treating children with diarrhea and planning preventive measures. Knowledge of the antimicrobial susceptibility of enteric bacterial pathogens will help healthcare workers provide evidence-based treatment for diarrheal disease. Therefore, the study provided information on the magnitude of diarrheal disease, the etiology of diarrhea among children, and the antimicrobial susceptibility of bacterial isolates. It will also serve as a stepping stone for other research that will be conducted in the future.

## Materials and methods

### Study area and study period

The study was carried out at Bule Hora Municipal Bule Hora General Hospital (BHGH) and the Bule Hora Health Centre was located in Bule Hora Town 467 km south of Addis Ababa. The capital of West Guji, Bule Hora, is located 1,716 m above sea level and has a latitude and longitude of 5035’N 38,015′E/5.5830 N 38.2500E. The average yearly rainfall is 648 mm. The city population center reports that as of 2007 E.C., 27,820 people were living there. A government general hospital and a health center are located in Bule Hora. The facilities provide services for an estimated 1,296,475 catchment population. The facilities provide outpatient care for children under 5 years of age years of age in one outpatient department (OPD). The study was conducted from May 2021 to July 2021.

### Study design and population

A cross-sectional study was conducted at Bule Hora General Hospital and Bule Hora Health Centre among children under 5 years of age who visited both health facilities with diarrhea. All under-five-year-old children who visited the under-five OPD with diarrhea during the study period were included in the study population.

### Inclusion and exclusion criteria

The study included all children under 5 years of age who came to health facilities with diarrhea during the study period and excluded children who had taken antibiotics 2 weeks before the data collection period, children in follow-up, and children under 5 years of age. Children were excluded whose guardians or parents were unwilling to participate.

### Sample size determination and sampling techniques

A single population proportion formula with 50% prevalence, a 95% confidence interval, and a 5% error margin was used to determine the sample size. The final sample size was 422 after 10% of the non-responding rate was taken into account. Children under 5 years of age whose guardians were willing to participate in the study and were available during the study period were recruited using consecutive sampling approaches until the desired sample size was reached.

### Data collection and laboratory procedure

Data on sociodemographic factors for diarrheal under five-year-old children were collected by a data collector who works under five OPD using a structured questionnaire by interviewing their caregivers or guardians. The questionnaire was pre-tested before commencing the data collection. A stool sample was collected for microscopy and culture. Stool samples were collected in a leak-proof stool cup from children with acute diarrhea with the help of a data collector and guardian. The isolation and characterization of intestinal parasites were performed by preparation of wet mounts and using the formal ether concentration technique. The identification of rotavirus was performed using a rotavirus antigen detection kit, and for bacteriological examination, the samples were transported to the Bule Hora University Microbiology Laboratory immediately after collection for processing in a sterile container. In the cases of samples delayed for more than 4 h, Cary-Blair transport medium (CA, USA) was used to preserve samples. Bacterial isolation was inoculated in the culture medium by streaking on Selenite F broth, MacConkey agar (Mac), eosin methylene Bule, deoxycholate citrate agar (DCA) and xylose-lysine deoxycholate (XLD) agar culture medium, and then plates were incubated at 37°C for 24–48 h. Using a common bacterial identification approach, the biochemical response pattern was used for confirmatory identification. The indole test, urease test, glucose test, maltose, lactose fermentation test, H2S, and motility test were used to biochemically analyze all suspected isolates according to conventional procedures to determine the important traits of the bacteria (17).

The Kirby-Bauer disc diffusion method was used to discover antimicrobial susceptibility patterns. Using a sterile cotton swab, the bacterial inoculum was evenly distributed on a sterile Mueller Hinton Agar Petri dish. To fully contact each disc with the agar surface, it must be pressed down. The plates were incubated for 18–24 h at 37°C, according to the recommendations of the Clinical Laboratory Standards Institute (CLSI) (18).

### Biochemical tests

Each colony on the plates was inspected following a standard operating procedure. For Gram-positive bacteria, catalase and coagulase tests were used. Tests such as triple sugar iron agar, oxidase, hydrogen sulfide production, gas production, motility, indole production, urease production, citrate utilization, and lysine decarboxylation and deamination were used for Gram-negative bacteria.

### Antimicrobial susceptibility testing

All Gram-positive and Gram-negative isolates underwent antimicrobial susceptibility tests on Muller-Hinton agar using the standard Kirby-Bauer diffusion technique under the guidelines set out by the Clinical and Laboratory Standards Institute (CLSI). There were nine types of antimicrobial discs used: Amoxicillin + clavulanic acid (AMC, 20/10 μg), Ampicillin (AMP, 10 μg), Cotrimoxazole (COT, 25 μg), Ceftriaxone (CRO, 30 μg), Chloramphenicol (CL, 30 μg), Ciprofloxacin (CIP, 5 μg), Gentamicin (GN, 10 μg), Norfloxacin (NOR, 5 μg), and Tetracycline (TE, 30 μg) (19).

### Data quality assurance

The standard operating procedure (SOP) was created according to the Ethiopian Public Health Institute (EPHI) guidelines and other textbooks. The SOP was closely adhered to during the manufacture of reagents and culture media, specimen processing, specimen culture, and microscopic examination. Before real data collection, a pre-test was carried out at Yabelo Hospital. Following the pretest, the required correction was made, and before entering the data, a timely and appropriate procedure was taken to ensure completeness. *The strains of Salmonella enteritidis* ATCC 13076, *E. coli* ATCC 25922, *P. aeruginosa* ATCC 27853, *S. aureus* ATCC 25923, *S. flexneri* ATCC 12022, and *E. faecalis* ATCC 929212 were used as quality control strains to ensure that the bacterial culture method met the CLSI requirements.

### Data management and analysis

The data was coded and then entered into Epi Data version 3.1. The data was then examined for consistency, completeness, and outliers using their distribution. Data that were inconsistent or lacking were removed from the analysis. Statistical Package Social Science software version 25.0 was used to analyze the data. We employ descriptive statistics of percentage frequency to characterize the study sample. Factors associated with the prevalence of enteropathogens were analyzed using binary logistic regression. Finally, the results are presented as tables and figures.

## Results

### Sociodemographic characteristics of the study participants

A total of 422 children under 5 years of age were involved in the study, with a response rate of 100%. The mean age of the children was 13 months, with a standard deviation of 4 months. Most of the children were between the ages of 13 and 24 months, 167 (39.6%). Almost half of the participants were born into a family size of less than or equal to three (49.5%). More than half of the children, 245 (58.1%), were urban residents. Regarding educational status, 94 (33.6%) of the mothers of the children had no formal education, 148 (35.07%), and 126 (29.5%) had primary education (Table 1).

**Table 1:** Sociodemographic characteristics of the study participants.

Variables	Category	Frequency	Percent
Sex	Male	224	53.1
	Female	198	46.9
Age groups (months)	0–6	72	17.06
	7–12	114	27.01
	13–24	167	39.57
	25–36	51	12.08
	37–48	16	3.8
	49–60	2	0.47
Family size	≤3	209	49.5
	4–6	180	42.7
	7–9	29	6.9
	≥10	4	0.9
Separate bedroom for children	Yes	68	16.1
	No	354	83.9
Educational status of guardian	No formal education	148	35.1
	Read and write	49	11.6
	Primary school education	126	29.9
	Secondary school education	61	14.5
	Higher education	58	13.7
Residence	Urban	245	58.1
	Rural	177	41.9
Family income	500–1,500	41	9.7
	1,501–2,500	209	49.5
	2,501–3,500	70	16.6
	>3,501	102	24.2

### Hygienic and sanitary conditions of the study participants children under 5 years of age

More than half of the 215 children in the study (50.9%) live in latrines, referring to their hygiene and sanitation conditions. Most of the mothers of the children wash their hands before feeding their children. Most of the children’s houses there are domestic animals living in house 290(68.7%) (Table 2).

**Table 2:** Hygienic condition of the children.

Variables	Positive (*n* = 72)	Negative (*n* = 350)
	No. (%)	No. (%)
Availability of latrine		
Yes	35(48.6)	180(51.4)
No	37(51.4)	170(48.6)
Maternal hand washing practice before feeding children		
Yes	38(52.8)	206(58.9)
No	34(47.2)	144(41.1)
Presence of domestic animal in the house		
Yes	60(83.3)	230(65.7)
No	12(16.7)	120(34.3)
Availability of tap water		
Yes	37(51.4)	184(52.6)
No	35(48.6)	166(47.4)

### Prevalence of enteropathogen

**Figure 1:**
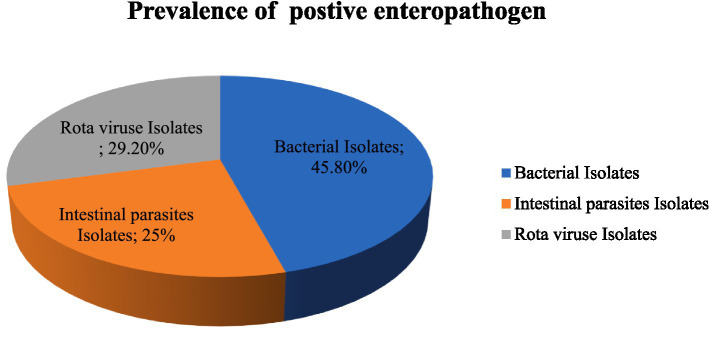
Prevalence of positive enteropathogen detected in under five-year-old children with a compliant range of diarrhea in Bule hora city, West Guji, Ethiopia,2021.

Of the 422 stool samples examined, 17.06% (72/422) tested positive for enteropathogens, with no mixed enteropathogens detected. Most of the enteropathogen isolates were bacterial, representing 7.8% (33/422) of the total samples, while 5% (18/422) were positive for rotavirus and 4.2% (21/422) for parasites. Among the detected enteropathogens, bacterial isolates were the most common at 45.8% (33/72), followed by rotaviruses at 29.2% (21/72) and enteric parasites at 25% (18/72) (Figure 1).

Among the parasites, *Entamoeba histolytica* was predominant, with a detection rate of 8/18 (44.4%), followed by *Giardia lamblia* 22.2% (4/18), *Hymnoleps nana* 16.7% (3/18) and others 16.7% (3/18). In the case of bacterial isolates, salmonella was the most predominant, accounting for more than half 16 (48.5%) of the cases, while each *Shigella* spp. and *E. coli* represented 18.18% (6/33), *Campylobacter jejuni* 9.09% (33/33) and others were 6.06%.

### Factors associated with the presence of enteropathogen

Most of the participants in this study, 396 (93.8%), drank unprotected water, and 324 (76.8%) of the carriers did not wash their hands properly. Of the carriers, about 231 (56.2%) maintained good hygiene, while 321 (78.1%) did not wash their children’s utensils. Only 17.2% of mothers and carriers; traditional pit latrines. When the associated factors for diarrhea in children were evaluated, it was found that the presence of enteropathogens in stool samples was statistically significant for an unprotected drinking water source (*p* = 0.002), poor hand washing practices of carriers (*p* = 0.02), and poor cleaning of utensils for child feeding (*p* = 0.025). The sociodemographic information of guardians and the existence of enteropathogens in the feces of children under 5 years of age did not differ statistically significantly. The sociodemographic information of the guardians is broken down in detail below. The presence of enteropathogens in the stool did not differ statistically significantly according to sex (*p* = 0.879).

### Antibiotic susceptibility patterns

In terms of antimicrobial susceptibility, six isolates of the *Shigella species* were shown to respond to ceftriaxone and ciprofloxacin; however, all strains showed resistance to ampicillin and Amoxicillin antibiotics. Among patients infected with *Salmonella* spp., 10, 8, 7, and 5 of the isolates were resistant to Amox/clav, chloramphenicol, tetracycline, and gentamycin; nearly all patients were resistant to ampicillin and amoxicillin. However, ceftriaxone and ciprofloxacin were effective against all isolates of Salmonella indicates a minor resistance to salmonella by norfloxacin and cotrimoxazole. Most *E. coli* and *C. jejuni* isolates showed susceptibility to all drugs tested in Table 3.

**Table 3:** Antimicrobial susceptibility patterns of bacteria isolates among children under 5 years of age in the city of Bule Hora, West Guji, Ethiopia.

Variables	*Salmonella* spp. (*n* = 16)		*Shigella* spp. (*n* = 6)		*E. coli* (*n* = 6).		*C. jejuni* (*n* = 3)	
Antibiotics	S	R	S	R	S	R	S	R
Ampicillin	0	16	0	6	3	3	1	2
Amox/clav	6	10	0	6	5	1	2	1
Chloramphenicol	8	8	2	4	6	0	3	0
Ciprofloxacin	16	0	6	0	6	0	3	0
Tetracycline	9	7	2	4	5	1	2	1
Ceftriaxone	16	0	6	0	6	0	3	0
Gentamycin	11	5	4	2	6	0	3	0
Norfloxacin	14	2	1	5	6	0	3	0
Cotrimoxazole	15	1	2	4	6	0	2	1

n, number of isolates; I, susceptible; R, Resistance.

### Rotavirus antigen test results

Children with watery diarrhea have a high percentage of positivity for the rotavirus, with 76.2% of the positive children having watery diarrhea (Table 4).

**Table 4:** Rota virus antigen test result.

Types of diarrhea	Positive (*n* = 21)	Negative (*n* = 401)
	No. (%)	No. (%)
Loose	1(4.7)	87(21.69)
Watery	16(76.2)	124(30.9)
Mucoid	0(0)	30(7.48)
Bloody and mucoid	2(9.5)	73(18.2)
Others	2(9.5)	87(21.7)

### Results of bacterial culture and types of diarrhea

Most patients with positive bacterial cultures experience watery diarrhea in 15 cases (45.4%), followed by loose diarrhea in 8 cases (24.2%), and bloody and mucoid diarrhea in 6 cases (18.18%) (Table 5).

**Table 5:** Results of bacterial isolates and types of diarrhea.

Variable Types of diarrhea	Positive (*n* = 33)	Negative (*n* = 389)
	No. (%)	No. (%)
Loose	8(24.2)	96(24.7)
Watery	15(45.4)	151(38.8)
Mucoid	3(9.09)	71(18.25)
Bloody and mucoid	6(18.18)	49(12.6)
Others	1(3.03)	22(5.65)

## Discussion

As one of the most common disorders in children under 5 years of age is still diarrhea, enteropathogens are important etiologic agents of this disease. This study looked at the antibiotic susceptibility patterns of bacterial isolates, as well as the loads of parasitic and bacterial agents on children with diarrhea. This study revealed that the overall prevalence of enteropathogens was 17.06% (72/422), which means that 7.8% were bacterial isolates, 4.2% were intestinal parasites, and 5% were Rotavirus.

Studies conducted in different parts of the world reveal considerable variance in the prevalence of bacteria, with some reporting lower rates and others higher rates. According to the results of the current study, children aged 13 to 24 months are more affected by infectious diarrhoea, e-enteropathogenic bacteria, intestinal parasites, and rotavirus.

In this study, the most prevalent isolates were *Salmonella* spp., *Shigella* spp., and *E. coli*. The findings were consistent with the studies conducted at Turunesh Bejing Hospital (3) and Jimma Health Centre (5). Contrary to this study, there were no *E. coli* isolates in the study carried out at Hara Jimma Health Centre (20–22).

Diarrhea remains one of the major diseases in children under 5 years of age, and enteropathogens play an important role as etiologic agents. This study investigated the antibiotic susceptibility patterns of bacterial isolates, as well as the loads of parasitic and bacterial agents on children with diarrhea. Studies conducted in different parts of the world reveal considerable variance in the prevalence of bacteria, with some reporting lower rates and others higher rates. According to the results of the current study, children aged 13–24 months are more affected by infectious diarrhea caused by bacteria, intestinal parasites, and the Rotavirus.

The achievement of the Sustainable Development Goals and advances in health and development are increasingly threatened by antimicrobial resistance. Antimicrobial resistance refers to the ability of bacterial, viral, parasitic, and fungal pathogens to withstand the effects of antibiotics that were once useful in the treatment of infections. It develops naturally over time, but is accelerated by the following: antimicrobial residues in soil, crops, and water; improper use of antimicrobial medications in the food, animal, health, and agriculture sectors; and lack of access to health services, particularly diagnostics and laboratory capacity (23).

The results of this study showed that almost all bacterial isolates were resistant to ampicillin and could also be resistant to amoxicillin. Each bacterial isolate had a distinct antimicrobial profile; *Shigella* spp. was the most resistant, followed by *Salmonella* spp., both of which exhibited significant rates of resistance to the antimicrobials examined. Amoxicillin and ampicillin showed the highest rates of antimicrobial resistance. This outcome is consistent with research done in Jimma, Nigeria, and India (10, 12, 20).

In the study area, factors such as drinking water from unprotected sources, poor hand washing practices of carriers, and inadequate cleaning of utensils used for child feeding were found to be significantly associated with the prevalence of enteropathogens among children under 5 years of age. This finding is supported by previous studies conducted in Ambo, Yaya Gulele, and Debre Berhan (17, 18, 24). The possible reason for this association is that parasites, enteric bacteria, and rotavirus present on hands, utensils or in unprotected water sources can easily be transmitted to children if adequate hygiene measures are not observed (25–27).

## Conclusion and recommendations

Both *Shigella* spp and *Salmonella* spp, isolates, were highly susceptible to ciprofloxacin and ceftriaxone, which are prescribed for diarrhoeal diseases caused by these pathogens in children, however, they showed a high rate of resistance to commonly used medications such as ampicillin and amoxicillin. Intestinal parasites such as *Entamoeba histolytica* were predominant, followed by *Giardia lamblia and Hymnoleps nana*. Rotavirus was the second predominant enteric pathogen. Drinking unsafe water, inadequate hand hygiene and the use of unclean feeding utensils have been associated with the occurrence of enteropathogens. Therefore, it is crucial to implement hygiene and sanitation measures to prevent infections in children under 5 years of age. In addition, antimicrobial susceptibility tests are recommended before prescribing treatments for children with diarrhea.

## Data Availability

The raw data supporting the conclusions of this article will be made available by the authors, without undue reservation.
